# *SEPP1* Influences Breast Cancer Risk among Women with Greater Native American Ancestry: The Breast Cancer Health Disparities Study

**DOI:** 10.1371/journal.pone.0080554

**Published:** 2013-11-20

**Authors:** Andrew J. Pellatt, Roger K. Wolff, Esther M. John, Gabriela Torres-Mejia, Lisa M. Hines, Kathy B. Baumgartner, Anna R. Giuliano, Abbie Lundgreen, Martha L. Slattery

**Affiliations:** 1 University of Utah, Department of Medicine, Salt Lake City, Utah, United States of America; 2 Cancer Prevention Institute of California, Fremont, California, United States of America; 3 Division of Epidemiology, Department of Health Research and Policy and Stanford Cancer Institute, Stanford University School of Medicine, Stanford, California, United States of America; 4 Instituto Nacional de Salud Pública, Centro de Investigación en Salud Poblacional, Ahuacatitlán, Cuernavaca Morelos, México; 5 University of Colorado at Colorado Springs, Department of Biology, Colorado Springs, Colorado, United States of America; 6 Department of Epidemiology and Population Health, School of Public Health & Information Sciences, James Graham Brown Cancer Center, University of Louisville, Louisville, Kentucky, United States of America; 7 Moffitt Cancer Center and Research Institute, Tampa, Florida, United States of America; Ohio State University Medical Center, United States of America

## Abstract

Selenoproteins are a class of proteins containing a selenocysteine residue, many of which have been shown to have redox functions, acting as antioxidants to decrease oxidative stress. Selenoproteins have previously been associated with risk of various cancers and redox-related diseases. In this study we evaluated possible associations between breast cancer risk and survival and single nucleotide polymorphisms (SNPs) in the selenoprotein genes *GPX1*, *GPX2*, *GPX3, GPX4, SELS, SEP15, SEPN1, SEPP1, SEPW1, TXNRD1*, and *TXNRD2* among Hispanic/Native American (2111 cases, 2597 controls) and non-Hispanic white (NHW) (1481 cases, 1586 controls) women in the Breast Cancer Health Disparities Study. Adaptive Rank Truncated Product (ARTP) analysis was used to determine both gene and pathway significance with these genes. The overall selenoprotein pathway P_ARTP_ was not significantly associated with breast cancer risk (P_ARTP_ = 0.69), and only one gene, *GPX3*, was of borderline significance for the overall population (P_ARTP_ =0.09) and marginally significant among women with 0-28% Native American (NA) ancestry (P_ARTP_=0.06). The *SEPP1* gene was statistically significantly associated with breast cancer risk among women with higher NA ancestry (P_ARTP_=0.002) and contributed to a significant pathway among those women (P_ARTP_=0.04). *GPX1, GPX3*, and *SELS* were associated with Estrogen Receptor-/Progesterone Receptor+ status (P_ARTP_ = 0.002, 0.05, and 0.01, respectively). Four SNPs (*GPX3* rs2070593, rs*GPX4* rs2074451, *SELS* rs9874, and *TXNRD1* rs17202060) significantly interacted with dietary oxidative balance score after adjustment for multiple comparisons to alter breast cancer risk. *GPX4* was significantly associated with breast cancer survival among those with the highest NA ancestry (P_ARTP_ = 0.05) only. Our data suggest that *SEPP1* alters breast cancer risk among women with higher levels of NA ancestry.

## Introduction

Selenium is a trace element essential for essential health that has been suggested to play a preventive role for a variety of chronic diseases, including various cancers [1-6]. This is likely due to the role of selenium as a constituent of various proteins, known as the selenoproteins [2,7]. Selenoproteins are a class of approximately 25 proteins containing a selenocysteine (SEC) residue. SEC is a cysteine analogue, where sulfur has been replaced by a selenium atom, synthesized from serine bound to a tRNA [8]. The incorporation of SEC is a complex process requiring an in-frame UGA codon, occurring as a stop codon, which is recognized as a SEC codon with the aid of a stem loop called a SEC insertion sequence (SECIS) and several other trans-acting factors [8,9]. 

The known human selenoproteins include the glutathione peroxidases (GPX), thioredoxin reductases (TXNRD), iodothyronine deiodinases (DIO), and selenophosphate synthetases 2 (SPS2) [10]. The other identified selenoproteins include, but are not limited to, selenoprotein n (SEPN1), selenoprotein p (SEPP1), selenoprotein s (SELS), selenoprotein w (SEPW1), and a 15-kDA selenoprotein (SEP15) [4,10]. Not all identified selenoproteins have been characterized; however, many of the selenoproteins with known function (including the GPXs, TXNRDs, SPS2, and DIOs), have redox functions [10] and behave as antioxidants [1] to reduce oxidative stress. 

In this study we evaluated single nucleotide polymorphisms (SNPs) in several selenoprotein coding genes for an association with breast cancer: glutathione peroxidase 1 (*GPX1*), glutathione peroxidase 2 (*GPX2*), glutathione peroxidase 3 (*GPX3*), glutathione peroxidase 4 (*GPX4*), *SELS, SEP15, SEPN1, SEPP1, SEPW1*, thioredoxin reductase 1 (*TXNRD1*), and thioredoxin reductase 2 (*TXNRD2*). These selenoproteins were selected for analysis because their functions have been characterized and many of them have been associated with risk of various types of cancer and/or oxidative stress [2-7,9,11-14]. The glutathione peroxidases (GPX1, GPX2, GPX3, and GPX4) primarily function to reduce oxidative stress by detoxifying hydrogen peroxide and other organic peroxides [2,13]. SELS is involved in inflammatory response [10]. SEP15 is a protein located primarily in the endoplasmic reticulum and plays a role in protein folding [2]. SEPN1 plays a role in redox homeostasis and protects against oxidative stress [15]. SEPP1 acts as a selenium transport protein, a heavy-metal chelator, and an antioxidant [10]. SEPW1 is a highly conserved protein that acts as an antioxidant protecting against oxidative stress [10]. The thioredoxin reductases (TXNRD1 and TXNRD2) are antioxidants that reduce the oxidized form of thioredoxin, an important regulator of redox-controlled cell functions and redox balance [12]. While many of these selenoprotein-coding genes have been associated with certain cancers, their association with breast cancer remains unclear.

In addition to evaluating SNPs in these genes for an association with breast cancer risk and survival, we also evaluated associations by level of Native American (NA) genetic ancestry. Higher levels of NA ancestry have been associated with reduced breast cancer risk [16,17] and serum selenium concentration has been shown to differ amongst racial and ethnic groups [18]. We also evaluated breast cancer associations by menopausal status and estrogen receptor (ER) and progesterone receptor (PR) status. Additionally, we evaluated breast cancer associations by dietary oxidative balance score (DOBS) since selenoprotein genes may interact with dietary factors that influence oxidative stress. 

## Methods

### Ethics Statement

All participants signed informed written consent prior to participation and each study was approved by the Institutional Review Board for Human Subjects at the participating institutions: University of Utah, University of Arizona, University of Colorado, University of New Mexico, Comisión de ética, and Institutional Review Board of the Cancer Prevention Institute of California. 

### Study Design

The Breast Cancer Health Disparities Study includes participants from three population-based case-control studies, the 4-Corners Breast Cancer Study, the Mexico Breast Cancer Study, and the San Francisco Bay Area Breast Cancer Study [17] who completed an in-person interview and who had a blood or mouthwash sample available for DNA extraction. In the 4- Corners Breast Cancer Study, participants were between 25 and 79 years of age with a histological confirmed diagnosis of *in situ* (n=341) or invasive (n=1492) cancer between October 1999 and May 2004; controls were selected from the target populations of cases living in Arizona, Colorado, New Mexico, and Utah and were frequency matched to cases on ethnicity and 5-year age distribution [19]. Participants from the Mexico Breast Cancer Study were between 28 and 74 years of age. Eligible cases in Mexico were women diagnosed with either a new histologically confirmed *in situ* or invasive breast cancer between January 2004 and December 2007 at 12 participating hospitals from three main health care systems; controls were randomly selected from the catchment area as the cases and frequency matched to cases based on 5-year age distribution, membership in health care institution, and place of residence. The San Francisco Bay Area Breast Cancer Study included women aged 35 to 79 years from the San Francisco Bay Area diagnosed with a first primary histologically confirmed invasive breast cancer between April 1995 and April 2002; controls were identified by random-digit dialing and frequency-matched to cases based on the expected race/ethnicity and 5-year age distribution [20,21]. 

### Data Harmonization

Data were harmonized across all study centers and questionnaires as previously described [17]. Women were classified as either pre-menopausal or post-menopausal based on responses to questions on menstrual history. Women who reported still having periods during the referent year (defined as the year before diagnosis for cases or before selection into the study for controls) were classified as pre-menopausal. Center-specific definitions were used to define post-menopausal women. Women were classified as post-menopausal if they reported either a natural menopause or if they reported taking hormone therapy and were still having periods or were at or above the 95th percentile of age for those who reported having a natural menopause (i.e., > 12 months since their last period. This age at menopause was site-specific by ethnicity: 58 years for NHW and 56 for Hispanic/Native women from the 4-Corners’ Breast Cancer Study; 54 for the Mexico Breast Cancer Study; and 55 for NHW and 56 for Hispanic women from the San Francisco Bay Area Breast Cancer Study. 

A dietary oxidative balance score (DOBS) that included nutrients with anti- or pro-oxidative balance properties was developed as previously reported [22]. Anti-oxidants included in the score were vitamin C, vitamin E, beta carotene (data for beta carotene were not available for Mexico), folic acid, and dietary fiber; alcohol was treated as a pro-oxidant. Nutrients per 1000 calories were evaluated and quartiles of intake were based on study-specific distributions; Long-term alcohol consumption was classified into three levels: the top 25^th^ percentile of consumption, all other drinkers, and non-drinkers. Referent year alcohol consumption was used for those women who did not have long-term alcohol measurements. In creating the DOBS, participants were assigned values of zero for low levels (first quartile) of exposure to anti-oxidants or high exposure to pro-oxidants (fourth quartile), one for intermediate levels (second and third quartiles) of exposure, and two for high levels (fourth quartile) of exposure to anti-oxidants and low exposure (first quartile) to pro-oxidants. 

### Genetic Data

DNA was extracted from either whole blood (n=7287) or mouthwash (n=634) samples. Whole Genome Amplification (WGA) was applied to the mouthwash-derived DNA samples prior to genotyping. A tagSNP approach was used to characterize variation across candidate genes. TagSNPs were selected using the following parameters: linkage disequilibrium (LD) blocks were defined using a Caucasian LD map and an r^2^=0.8; minor allele frequency (MAF) >0.1; range= -1500 bps from the initiation codon to +1500 bps from the termination codon; and 1 SNP/LD bin. We distinguished European and NA ancestry in the study population by using 104 Ancestry Informative Markers (AIMs) [17]. A multiplexed bead array assay format based on GoldenGate chemistry (Illumina, San Diego, California) was used for genotyping. A genotyping call rate of 99.93% was attained (99.65% for WGA samples). We included 132 blinded internal replicates representing 1.6% of the sample set.  The duplicate concordance rate was 99.996% as determined by 193,297 matching genotypes among sample pairs.  In the current analysis we evaluated *GPX1* (2 SNPs), *GPX2* (4 SNPs), *GPX3* (3 SNPs), *GPX4* (1 SNP)*, SEPP1* (2 candidate SNPs and 1 tagSNP)*, SELS* (2 SNPs), *SEP15* (4 SNPs), *SEPN1* (5 SNPs), *SEPW1* (3 SNPs), *TXNRD1* (7 SNPs), and *TXNRD2* (20 SNPs). A description of these genes and SNPs is shown in online Table S1. *SEP15* rs9433110 was not analyzed since it was not in Hardy-Weinberg Equilibrium (HWE) among NHW participants. Online Table S2 shows minor allele frequency (MAF) and HWE by ancestry groups. It should be noted that in most instances a trend in a different prevalence of MAF across ancestry groups was noted and in some instances, such as *SEPP1* rs6865453, we observed a reversal in the major and minor allele from the most European to the most Native ancestry groups.

### Tumor Characteristics and Survival

Data for survival and ER/PR tumor status were available for cases from the 4-Corners Breast Cancer Study and the San Francisco Bay Area Breast Cancer Study. 

Information on stage at diagnosis, months of survival after diagnosis, cause of death, and ER and PR status were available from cancer registries in Utah, Colorado, Arizona, New Mexico, and California. Information on ER and PR status of tumors was available for 1019 (69%) NHW and 977 (75%) Hispanic/NA cases. Surveillance Epidemiology and End Results (SEER) summary disease stage, based on three codes of local, regional, and distant, was used. Data on survival and ER and PR tumor status were not available from the Mexico Breast Cancer Study. 

### Statistical Methods

#### Genetic Ancestry Estimation

The program STRUCTURE was used to compute individual ancestry for each study participant assuming two founding populations [23,24]. A three-founding population model was assessed but did not fit the population structure with the same level of repeatability and correlation among runs as the two-founding population model. Participants were classified by level of percent NA ancestry. Assessment across categories of ancestry was done using cut-points based on the distribution of genetic ancestry in the control population. These cut-points, 0-28%, >28-70%, and >70-100%, maximized power within all three ancestry groups to assess ancestry-specific associations. Genetic ancestry was used as a continuous variable when included in the models to adjust for possible confounding. 

#### SNP Associations

Genes and SNPs were assessed for their association with breast cancer risk by strata of menopausal status and genetic ancestry in the whole population and by ER/PR status for the 4-Corners Breast Cancer Study and the San Francisco Bay Area Study. Statistical analyses were performed using SAS version 9.3 (SAS Institute, Cary, NC) unless otherwise noted. Logistic regression models were used to estimate odds ratios (OR) and 95% confidence intervals (CI) for breast cancer risk associated with SNPs, adjusting for age, study center, genetic ancestry, body mass index (BMI of kg/m^2^) during referent year, and parity. The generalized logit link function was used when estimating breast cancer risk by ER/PR status. Associations with SNPs were assessed assuming co-dominant models. Based on the initial assessment, SNPs which appeared to have a dominant or recessive mode of inheritance were evaluated with those inheritance models in subsequent analyses. Stratified analyses tests for interactions were calculated using a 1 degree of freedom (df) Wald chi-square tests; p values based on 4-df Wald tests measure the overall SNP (treated as continuous) association with breast cancer risk by ER/PR status. Adjustments for multiple comparisons within the gene used the step-down Bonferroni correction (i.e., Holm method) taking into account the correlated nature of the data using the SNP spectral decomposition method proposed by Nyholt [25] and modified by Li and Ji [26]. 

#### Survival Analysis

Survival months were calculated based on month and year of diagnosis and month and year of death or date of last contact by the cancer registries. Associations between SNPs and risk of dying of breast cancer among primary invasive cases were evaluated using Cox proportional hazards models to obtain multivariate hazard ratios (HR) and 95% CI among all cases and by genetic ancestry strata. The upper two ancestry strata were combined to evaluate survival by ancestry groups since survival data were not available for the Mexico study site. In the analysis of breast cancer survival, individuals were censored when they died of causes other than breast cancer or were lost to follow-up. In addition to the minimal adjustments for age, study center, genetic ancestry, BMI during referent year, and parity, models were also adjusted for SEER summary stage. 

#### ARTP Analysis

We used the adaptive rank truncated product (ARTP) method that is based on a highly efficient permutation algorithm to determine the significance of association of each gene and of the pathway with breast cancer overall, by genetic ancestry, and by ER/PR strata. The gene p values were generated using the ARTP package in R, permuting outcome status 10,000 times while adjusting for age, BMI during referent year, and genetic ancestry [27,28]. The ARTP method was also applied to survival data using Cox proportional hazard models in R with an additional adjustment for SEER summary stage to generate p values based on likelihood ratio tests. The survival outcome (i.e., vital status and survival months) was permuted 10,000 times in R. We report both pathway and gene p values (P_ARTP_). 

## Results

The overall selenoprotein pathway P_ARTP_ was not significantly associated with breast cancer risk (P_ARTP_ = 0.69), and the association with only one gene, *GPX3*, was of borderline significance for the entire population (P_ARTP_ =0.09). However, we observed several significant associations prior to adjustment for multiple comparisons between selenoprotein SNPs and breast cancer risk (Table 1). *GPX3* rs8177447 (p=0.009, p_adj_=0.02) was associated with breast cancer risk for the entire population. *GPX3* was marginally significant among individuals with ≤28% NA ancestry (P_ARTP_ =0.06) and *SEPP1* was significant among those with >28% NA ancestry (P_ARTP_ =0.002). The overall pathway was statistically significant (P_ARTP_ =0.04) among women with higher NA ancestry mainly because of the strong association with *SEPP1* and breast cancer risk. *GPX3* rs8177447 (p=0.014, p_adj_ =0.035) and *SELS* rs4965814 (p=0.046, p_adj_ =0.06) were significant among individuals of <28% NA ancestry; *SEPP1* rs230812 (p=0.025, p_adj_ = 0.04), and *SEPP1* rs6865453 (p=0.002, p_adj_ = 0.005) were associated with breast cancer risk in individuals of 28-70% NA ancestry; and *SEPN1* rs718391 (p=0.02, p_adj_ =0.09) was marginally associated with breast cancer risk among women of >70% NA ancestry. Associations were similar for women with >28% NA ancestry for *SEPP1* rs230812 and rs6865453; when combining the upper ancestry groups these two SNPs were statistically significant (OR=1.33, 95% CI 1.09,1.64 and OR=0.80, 95% CI 0.66,0.96, respectively). Despite apparent differences between SNP associations and breast cancer risk among different NA ancestry groups, only *SEPN1* rs718391 (p=0.03 p_adj_ =0.10), *SEPP1* rs230812 (p=0.005, p_adj_ = 0.008), and *SEPP1* rs6865453 (p=<0.001, p_adj_ = 0.002) showed significant interaction by ancestry. 

**Table 1:** Associations between selenoprotein genes and breast cancer risk by genetic ancestry.

		Everyone						0 - 28% Native American Ancestry											>28 - 70% Native American Ancestry												>70 - 100% Native American Ancestry									Interaction P-value				
				Controls	Cases	OR^1^	(95% CI)	P_ARTP_		Controls		Cases	OR^1^			(95% CI)				P_ARTP_		Controls		Cases		OR^1^		(95% CI)				P_ARTP_	Controls		Cases		OR^1^	(95% CI)		P_ARTP_				
	*GPX3* (rs8177447)							0.09										0.06												0.67										0.53	0.38			
	CC/CT			4071	3469	1.00				1798	1675		1.00									1648		1359		1.00							625		435		1.00							
	TT			78	100	1.50	(1.11,	2.03)		44	67		1.63	(1.11,		2.40)						30		31		1.34		(0.80,		2.24)			4		2		0.72		(0.13,	4.05)				
P-value (raw; adjusted)0.009, 0.02													0.014, 0.035													0.27, 0.63											0.71, 1.00							
*SELS* (rs4965814)							0.70										0.43												0.31										0.58	0.14			
																										
	TT	2042	1833	1					1216	1136		1							616		559		1									210	138	1									
	TC	1665	1362	0.98	(0.89,	1.08)			564	522		0.99	(0.86,		1.14)				815		626		0.88		(0.75,		1.03)					286	214	1.21		(0.91,		1.61)					
	CC	443	373	1.05	(0.89,	1.23)			62	83		1.41	(1.00,		1.98)				248		205		0.93		(0.75,		1.17)					133	85	1.04		(0.73,		1.49)					
			P-value (raw; adjusted) 0.58, 0.98											0.050, 0.067											0.55, 0.90											0.83, 1.00							
*SEPN1* (rs718391)							0.46										0.27												0.73										0.16	0.034			
	CC/CG	3562	3024	1.00					1488	1369		1.00							1496		1239		1.00									578	416	1.00									
	GG	585	540	1.03	(0.91,	1.18)			354	372		1.13	(0.96,		1.33)				181		147		0.98		(0.78,		1.24)					50	21	0.54		(0.31,		0.92)					
			P-value (raw; adjusted) 0.62, 1.00											0.14, 0.29											0.89, 0.89											0.024, 0.09							
*SEPP1* (rs230812)^2^							0.16										0.57												0.01										0.15				
	AA	1456	1222	1.00					488	501		1.00							634		504		1.00									334	217	1.00						0.005			
	AC	1898	1574	0.96	(0.86,	1.06)			921	819		0.87	(0.75,		1.02)				755		603		0.99		(0.84,		1.16)					222	152	1.08		(0.82,		1.42)					
	CC	605	589	1.09	(0.95,	1.26)			360	336		0.90	(0.74,		1.10)				200		210		1.30		(1.03,		1.64)					45	43	1.45		(0.91,		2.30)					
		P-value (raw; adjusted) 0.21, 0.33												0.31. 0.79											0.025, 0.039											0.12, 0.16							
*SEPP1* (rs6865453)^3^																																								<.001			
	AA	1463	1387	1.00					897	834		1.00							481		476		1.00									85	77	1.00									
	AC/CC	2400	1937	0.90	(0.81,	0.99)			832	802		1.03	(0.90,		1.18)				1066		811		0.78		(0.66,		0.91)					502	324	0.73		(0.52,		1.04)					
			P-value (raw; adjusted) 0.037, 0.10											0.65, 1.00											0.002, 0.005											0.08, 0.16							
*TXNRD1* (rs4964287)							0.46										0.45												0.69										0.34	0.27			
	CC/CT	3884	3286	1.00					1672	1561		1.00							1597		1303		1.00									615	422	1.00									
	TT	264	283	1.20	(1.00,	1.43)			170	181		1.13	(0.91,		1.42)				81		87		1.27		(0.93,		1.75)					13	15	1.52		(0.70,		3.30)					
			P-value (raw; adjusted) 0.046, 0.23											0.26, 0.79											0.14, 0.65											0.29, 0.54							
*TXNRD2* (rs2073750)							0.25										0.69												0.54										0.33	0.99			
	GG	2169	1868	1.00					1087	1030		1.00							809		642		1.00									273	196	1.00									
	GA	1664	1413	1.02	(0.93,	1.12)			651	613		1.00	(0.87,		1.16)				732		607		1.06		(0.91,		1.23)					281	193	1.00		(0.77,		1.31)					
	AA	311	284	1.13	(0.95,	1.35)			104	96		0.97	(0.73,		1.30)				132		140		1.38		(1.06,		1.79)					75	48	1.00		(0.66,		1.52)					
			P-value (raw; adjusted) 0.17, 1.00											0.85, 1.00											0.018, 0.27											0.99, 1.00							
*TXNRD2* (rs5992493)																																								0.64			
	AA/AG	4057	3454	1.00					1789	1676		1.00							1646		1350		1.00									622	428	1.00									
	GG	92	115	1.42	(1.08,	1.88)			52	66		1.35	(0.93,		1.95)				33		40		1.43		(0.89,		2.30)					7	9	1.63		(0.59,		4.48)					
			P-value (raw; adjusted) 0.013, 0.20											0.12, 1.00											0.14, 1.00											0.35, 1.00							
*TXNRD2* (rs3788314)																																								0.33			
	GG	1158	941	1.00					532	489		1.00							443		340		1.00									183	112	1.00									
	GA	2071	1770	1.06	(0.95,	1.18)			910	838		0.98	(0.84,		1.15)				831		713		1.12		(0.94,		1.33)					330	219	1.09		(0.81,		1.47)					
	AA	918	853	1.14	(1.00,	1.30)			399	412		1.10	(0.92,		1.33)				403		335		1.08		(0.88,		1.33)					116	106	1.42		(0.99,		2.04)					
			P-value (raw; adjusted) 0.04, 0.61											0.31, 1.00											0.47, 1.00											0.055, 0.78							
*TXNRD2* (rs3788317)																																								0.34			
	GG	2356	1979	1.00					1094	1017		1.00							925		744		1.00									337	218	1.00									
	GT	1554	1355	1.04	(0.95,	1.15)			651	633		1.03	(0.90,		1.19)				656		539		1.03		(0.88,		1.20)					247	183	1.15		(0.88,		1.49)					
	TT	239	235	1.20	(0.99,	1.45)			97	92		1.02	(0.75,		1.37)				97		107		1.37		(1.02,		1.84)					45	36	1.23		(0.76,		1.99)					
			P-value (raw; adjusted) 0.07, 0.85											0.91, 1.00											0.039, 0.54											0.40, 1.00							

^1^ Odds Ratios (OR) and 95% Confidence Intervals (CI) adjusted for age, study, BMI during referent year, parity, and genetic ancestry (continuous).

^2^ Association for *SEPP1* rs230812 was 1.33 (95% CI 1.09,1.64; p = 0.006) for the CC genotype for women with >28% NA ancestry. pathway P_ARTP_ is 0.04

^3^ Association for *SEPP1* rs6865453 was 0.78 (95%CI 0.67,0.90; p<0.001) for AC/CC genotype for women with >28% NA ancestry. *SEPP1* P_ARTP_ for >28% NA ancestry is 0.002 and pathway P_ARTP_ is 0.04

Assessment of SNP associations by ER/PR status indicated that some SNPs were associated with breast cancer risk by ER/PR status; several genes had statistically significant P_ARTP_ values (Table 2). *GPX1, GPX3*, and *SELS* were associated with ER-/PR+ status (P_ARTP_ = 0.002, 0.05, and 0.01, respectively). *GPX1* rs1800668 was strongly associated with increased likelihood of ER-/PR+ phenotype p<0.001; p_adj_=0.002), while rs3448 was associated with a decreased likelihood of this phenotype (p=0.04). *GPX2* rs11623705 was associated with increased risk of ER-/PR+ (p=0.002; p_adj_ =0.006). *GPX3* rs8177447 showed a significant association with an increased likelihood of an ER-/PR+ phenotype (p=0.025; p_adj_ =0.07) as did *SELS* rs9874 (p=0.041; p_adj_=0.04) and *SELS* rs4965814 (p=0.01; p_adj_=0.02). *SELS* was also significantly associated with ER+/PR- tumors (P_ARTP_ = 0.009); *SELS* rs4965814 was positively associated with ER+/PR- (p=0.004; p_adj_=0.006). Multiple SNPs in *SEP15* showed an association with increased likelihood of ER+/PR- tumors: rs5859 (p=0.025; p_adj_=0.04), rs486133 (p=0.05; p_adj_=0.05), and rs1407131 (p=0.002; p_adj_=0.006). *TXNRD2* rs2073750 was marginally inversely associated with ER-/PR+ tumors (p=0.005; p_adj=_ 0.08) with an overall gene P_ARTP_ of 0.08. None of the genes evaluated showed a significant association with ER-/PR- tumor phenotype according to ARTP. 

**Table 2:** Associations between selenoprotein genes and breast cancer risk by ER/PR tumor status.

		Controls	ER+ / PR+					ER+ / PR-					ER- / PR+					ER- / PR-							
			N	N	OR^1^	(95% CI)		P_ARTP_	N	OR	(95% CI)		P_ARTP_	N	OR	(95% CI)		P_ARTP_	N	OR	(95% CI)		P_ARTP_	Multinomial p-value	
	*GPX1* (rs1800668)							0.94					0.74					0.002					0.54	0.007	
		CC	1608	638	1.00				107	1.00				12	1.00				198	1.00					
		CT	1117	425	0.91	(0.79,	1.06)		73	0.93	(0.68,	1.27)		19	2.60	(1.24,	5.47)		128	0.92	(0.73,	1.17)			
		TT	191	92	1.11	(0.85,	1.46)		20	1.43	(0.86,	2.39)		6	5.85	(2.08,	16.47)		19	0.81	(0.49,	1.33)			
	P-value (raw; adjusted)			0.44, 0.84					0.17, 0.32					<.001, 0.002					0.40, 0.75					
*GPX1* (rs3448)																							0.31	
	CC	1984	796	1.00				148	1.00				34	1.00				267	1.00					
	CT/TT	1181	502	1.01	(0.89,	1.16)		87	0.96	(0.72,	1.27)		9	0.46	(0.22,	0.96)		148	0.94	(0.75,	1.16)			
	P-value (raw; adjusted)			0.83, 0.84					0.77, 0.77					0.039, 0.039					0.55, 0.75					
*GPX2* (rs11623705)							0.31					0.71					0.30					0.23	0.61	
		GG	2500	1015	1.00				192	1.00				36	1.00				340	1.00				
	GT	628	271	1.05	(0.89,	1.23)		36	0.74	(0.51,	1.08)		4	0.44	(0.16,	1.26)		74	0.87	(0.67,	1.14)			
	TT	36	11	0.72	(0.36,	1.43)		7	2.35	(1.02,	5.42)		3	7.19	(2.06,	25.06)		1	0.22	(0.03,	1.63)			
	P-value (raw; adjusted)			0.35, 0.81					0.045, 0.13					0.002, 0.006					0.14, 0.26					
*GPX3* (rs8177447)							0.56					0.30					0.048					0.17	0.056	
	CC	2270	926	1.00				180	1.00				24	1.00				297	1.00					
	CT	827	323	0.92	(0.79,	1.07)		48	0.71	(0.51,	0.98)		16	1.86	(0.98,	3.56)		100	0.91	(0.72,	1.16)			
	TT	68	49	1.79	(1.23,	2.62)		7	1.38	(0.62,	3.08)		3	4.12	(1.19,	14.24)		18	1.94	(1.13,	3.31)			
	P-value (raw; adjusted)			0.003, 0.007					0.43, 0.68					0.025, 0.066					0.016, 0.04					
*SELS* (rs9874)							0.21					0.009					0.01					0.71	0.07	
	AA	2355	993	1.00				168	1.00				26	1.00				305	1.00					
	AG/GG	810	305	0.88	(0.76,	1.03)		67	1.15	(0.86,	1.55)		17	1.91	(1.03,	3.54)		109	1.03	(0.81,	1.30)			
	P-value (raw; adjusted)			0.10, 0.16					0.35, 0.35					0.04, 0.04					0.83, 0.99					
*SELS* (rs4965814)																							0.005	
	TT	1716	719	1.00				108	1.00				14	1.00				215	1.00					
	TC/CC	1450	579	1.02	(0.89,	1.17)		127	1.51	(1.15,	2.00)		29	2.37	(1.22,	4.59)		199	1.06	(0.85,	1.31)			
	P-value (raw; adjusted)			0.81, 0.81					0.004, 0.006					0.011, 0.017					0.62, 0.99					
*SEP15* (rs5859)																							0.87	
	CC	1878	721	1.00			0.37	131	1.00			0.84	23	1.00			0.77	218	1.00			0.92		
	CT	925	376	1.04	(0.89,	1.20)		54	0.82	(0.59,	1.14)		13	1.19	(0.60,	2.38)		111	1.03	(0.81,	1.31)			
	TT	109	53	1.23	(0.88,	1.73)		15	1.93	(1.09,	3.42)		1	0.85	(0.11,	6.37)		13	1.05	(0.58,	1.89)			
	P-value (raw; adjusted)			0.23, 0.63					0.025, 0.04					0.87, 1.00					0.88, 1.00					
*SEP15* (rs486133)																							0.86	
	TT	2138	862	1.00				160	1.00				30	1.00				288	1.00					
	TC	925	385	1.02	(0.88,	1.17)		61	0.88	(0.64,	1.19)		12	0.95	(0.48,	1.87)		114	0.93	(0.74,	1.17)			
	CC	103	51	1.19	(0.84,	1.69)		14	1.80	(1.00,	3.24)		1	0.76	(0.10,	5.64)		13	0.97	(0.53,	1.75)			
	P-value (raw; adjusted)			0.32, 0.63					0.05, 0.05					0.79, 1.00					0.91, 1.00					
*SEP15* (rs1407131)																							0.69	
	TT	2478	987	1.00				187	1.00				36	1.00				324	1.00					
	TC	648	293	1.11	(0.95,	1.30)		39	0.79	(0.55,	1.12)		6	0.64	(0.27,	1.54)		87	1.04	(0.80,	1.33)			
	CC	40	18	1.14	(0.65,	2.01)		9	3.21	(1.52,	6.79)		1	1.81	(0.24,	13.69)		4	0.78	(0.28,	2.21)			
	P-value (raw; adjusted)			0.64, 0.65					0.002, 0.006					0.575, 1.00					0.64, 1.00					
*TXNRD1* (rs5018287)							0.12					0.76					0.76					0.94	0.15	
	GG	913	414	1.00				68	1.00				11	1.00				121	1.00					
	GA	1571	631	0.87	(0.75,	1.01)		119	0.98	(0.72,	1.34)		21	1.11	(0.53,	2.32)		198	0.95	(0.75,	1.21)			
	AA	678	252	0.81	(0.67,	0.98)		48	0.92	(0.62,	1.35)		11	1.39	(0.60,	3.23)		96	1.09	(0.82,	1.45)			
	P-value (raw; adjusted)			0.030, 0.15					0.66, 1.00					0.45, 0.87					0.56, 1.00					
*TXNRD1* (rs7962759)																							0.41	
	CC	2233	873	1.00				163	1.00				32	1.00				289	1.00					
	CG	848	375	1.09	(0.94,	1.26)		66	1.05	(0.77,	1.42)		7	0.59	(0.26,	1.37)		113	1.04	(0.82,	1.31)			
	GG	85	50	1.43	(1.00,	2.06)		6	0.96	(0.41,	2.24)		4	3.60	(1.21,	10.73)		13	1.21	(0.66,	2.21)			
	P-value (raw; adjusted)			0.05, 0.21					0.92, 1.00					0.02, 0.11					0.54, 1.00					
*TXNRD2* (rs1044732)							0.36					0.93					0.08					0.80	0.20	
	AA	2228	884	1.00				148	1.00				35	1.00				264	1.00					
	AG/GG	689	271	0.96	(0.82,	1.13)		52	1.11	(0.80,	1.55)		2	0.19	(0.04,	0.78)		81	0.98	(0.75,	1.28)			
	P-value (raw; adjusted)			0.66, 1.00					0.54, 1.00					0.02, 0.30					0.88, 1.00					
*TXNRD2* (rs3788305)																							0.28	
	AA	944	423	1.00				70	1.00				16	1.00				129	1.00					
	AG/GG	2220	875	0.87	(0.75,	1.00)		165	1.00	(0.75,	1.34)		27	0.71	(0.38,	1.33)		286	0.94	(0.75,	1.17)			
	P-value (raw; adjusted)			0.045, 0.68					0.99, 1.00					0.28, 1.00					0.58, 1.00					
*TXNRD2* (rs2073750)																							0.09	
	GG	1713	707	1.00				125	1.00				32	1.00				223	1.00					
	GA/AA	1448	590	1.01	(0.89,	1.16)		108	1.03	(0.79,	1.35)		11	0.37	(0.19,	0.75)		191	0.99	(0.80,	1.22)			
	P-value (raw; adjusted)			0.84, 1.00					0.81, 1.00					0.005, 0.08					0.91, 1.00					

^1^ Odds Ratios (OR) and 95% Confidence Intervals (CI) adjusted for age, study, BMI during referent year, parity, and genetic ancestry (continuous); data from 4-CBCS and SFBCS

Five SNPs, *GPX3* rs2070593 (p=0.002), *GPX4* rs2074451 (p=0.046), *SELS* rs9874 (p=0.029), *TXNRD1* rs17202060 (p=0.007) and *TXNRD2* rs7322262 (p=0.025), significantly interacted with DOBS to alter breast cancer risk (Table 3). Of these SNPs, only *TXNRD2* rs732262 (p_adj_ =0.38) did not remain statistically significantly associated with breast cancer risk after multiple comparison adjustment. 

**Table 3:** Associations between dietary oxidative balance score (DOBS) and selenoprotein genes and risk of breast cancer.

		Dietary Oxidative Balance Score^1^																
		Quartile 1				Quartile 2				Quartile 3				Quartile 4				Interaction P-value
		Controls	Cases	OR^2^	(95% CI)	Controls	Cases	OR	(95% CI)	Controls	Cases	OR	(95% CI)	Controls	Cases	OR	(95% CI)	raw, adjusted
*GPX3* (rs2070593)																		0.002, 0.005
	GG	580	589	1.00		596	521	0.88	(0.75, 1.04)	695	542	0.79	(0.67, 0.93)	506	463	0.92	(0.78, 1.10)	
	GA/AA	350	369	1.08	(0.89, 1.30)	337	337	1.04	(0.86, 1.26)	508	381	0.79	(0.66, 0.94)	421	262	0.66	(0.54, 0.80)	
*GPX4* (rs2074451)																		0.046, 0.046
	GG/GT	741	730	1.00		759	630	0.87	(0.75, 1.01)	928	738	0.83	(0.72, 0.96)	705	562	0.84	(0.72, 0.97)	
	TT	144	147	1.00	(0.78, 1.29)	124	143	1.13	(0.87, 1.47)	199	121	0.61	(0.47, 0.78)	160	110	0.68	(0.52, 0.89)	
*SELS* (rs9874)																		0.029, 0.048
	AA	691	740	1.00		706	656	0.89	(0.76, 1.03)	926	717	0.75	(0.65, 0.86)	734	558	0.73	(0.62, 0.85)	
	AG	223	216	0.88	(0.71, 1.10)	215	186	0.81	(0.64, 1.01)	270	192	0.67	(0.54, 0.83)	180	152	0.81	(0.63, 1.03)	
	GG	16	6	0.34	(0.13, 0.88)	18	19	0.95	(0.49, 1.83)	10	16	1.45	(0.65, 3.24)	17	18	1.02	(0.52, 2.00)	
*TXNRD1* (rs17202060)																		0.007, 0.034
	CC/CT	763	841	1.00		788	721	0.85	(0.74, 0.98)	1018	778	0.71	(0.62, 0.82)	781	605	0.72	(0.62, 0.83)	
	TT	165	121	0.68	(0.52, 0.88)	152	140	0.86	(0.67, 1.11)	188	147	0.76	(0.60, 0.97)	150	123	0.81	(0.62, 1.05)	
*TXNRD2* (rs732262)																		0.025, 0.378
	GG	663	644	1.00		681	566	0.87	(0.75, 1.02)	821	633	0.82	(0.70, 0.95)	612	507	0.87	(0.74, 1.02)	
	GA	207	210	1.08	(0.86, 1.35)	182	187	1.14	(0.90, 1.44)	276	206	0.82	(0.66, 1.02)	233	150	0.73	(0.58, 0.93)	
	AA	15	23	1.78	(0.91, 3.46)	20	20	1.14	(0.60, 2.16)	30	20	0.75	(0.42, 1.34)	20	15	0.83	(0.42, 1.64)	

^1^ DOBS consists of alcohol (pro-oxidant), vitamins C and E, beta carotene, folic acid, and dietary fiber (anti-oxidants).

^2^ Odds Ratios (OR) and 95% Confidence Intervals (CI) adjusted for age, genetic ancestry, study center, BMI during referent year, and parity.

Multiple genes and SNPs in our analysis showed an association with breast cancer survival (Table 4). *GPX4* was significantly associated with better breast cancer survival among those with the highest NA ancestry (P_ARTP_ = 0.05). *GPX4*_*GT/TT*_ rs2074451 showed a marginal inverse association with survival for individuals among women with >28% NA ancestry (p=0.055; p_adj_ = 0.055; p interaction=0.06). Several SNPs in *TXNRD2* were associated with survival prior to adjustment for multiple comparisons. Additionally, *TXNRD2*_*AA*_ rs3788314 and *TXNRD2*_*CT/TT*_ rs4333017 showed significant interaction across genetic ancestry (p_int_=0.035 and p_int_=0.017, respectively).

**Table 4:** Association between selenoprotein genes and survival by ancestry.

		Overall							0 - 28% Native American Ancestry										29 - 100% Native American Ancestry								
		Death/Person Years	HR^1^	(95% CI)		P_ARTP_		Death/Person Years		HR		(95% CI)			P_ARTP_			Death/Person Years		HR		(95% CI)			P_ARTP_		Interaction P-value	
*GPX4* (rs2074451)							0.46									0.55d										0.05	0.06
	GG	69 / 5878	1.00						36 / 3476		1.00								33 / 2402		1.00						
	GT/TT	127 / 12780	0.90	(0.67,	1.21)				91 / 8514		1.13		(0.76,	1.67)					36 / 4265		0.62		(0.39,	1.01)			
	P-value (raw, adjusted) 0.50, 0.50										0.55, 0.55										0.06, 0.06						
*SEPW1* (rs3786777)							0.28									0.06										0.81	0.13
	TT	57 / 6219	1.00						26 / 3389		1.00								31 / 2830		1.00						
	TG/GG	169 / 15030	1.28	(0.94,	1.73)				107 / 9331		1.65		(1.07,	2.54)					62 / 5700		0.99		(0.64,	1.53)			
	P-value (raw, adjusted) 0.11, 0.21										0.02, 0.04										0.97, 0.97						
*TXNRD2* (rs9606173)							0.24									0.76										0.09	0.63
	AA	168 / 14566	1.00						105 / 9429		1.00								63 / 5137		1.00						
	AT/TT	61 / 6816	0.73	(0.54,	0.99)				30 / 3355		0.80		(0.53,	1.20)					31 / 3462		0.68		(0.44,	1.06)			
	P-value (raw, adjusted) 0.04, 0.57										0.28, 1.00										0.09, 0.97						
*TXNRD2* (rs732262)																											0.15
	GG	164 / 14336	1.00						111 / 10086		1.00								53 / 4250		1.00						
	GA/AA	32 / 4322	0.63	(0.43,	0.94)				16 / 1905		0.87		(0.52,	1.48)					16 / 2417		0.47		(0.27,	0.83)			
	P-value (raw, adjusted) 0.02, 0.33										0.62, 1.00										0.01, 0.15						
*TXNRD2* (rs3788314)																											
	GG	69 / 5595	1.00						39 / 3530		1.00								30 / 2065		1.00						0.035
	GA	108 / 10911	0.86	(0.63,	1.16)				58 / 6296		0.86		(0.57,	1.30)					50 / 4614		0.84		(0.52,	1.34)			
	AA	51 / 4836	0.91	(0.63,	1.31)				38 / 2931		1.26		(0.80,	1.99)					13 / 1905		0.50		(0.26,	0.97)			
	P-value (raw, adjusted) 0.61, 1.00 .										0.31, 1.00										0.04, 0.52						
*TXNRD2* (rs3788317)																											0.04
	GG	133 / 11991	1.00						74 / 7409		1.00								59 / 4582		1.00						
	GT	84 / 8163	0.94	(0.71,	1.24)				51 / 4744		1.04		(0.72,	1.49)					33 / 3419		0.82		(0.53,	1.26)			
	TT	12 / 1228	0.86	(0.48,	1.56)				10 / 630		1.58		(0.81,	3.07)					2 / 598		0.27		(0.06,	1.10)			
	P-value (raw, adjusted) 0.62, 1.00										0.18, 1.00										0.07, 0.81						
*TXNRD2* (rs4333017)																											
	CC	180 / 16994	1.00						107 / 9640		1.00								73 / 7354		1.00						0.017
	CT/TT	49 / 4388	1.13	(0.82,	1.56)				28 / 3143		0.85		(0.56,	1.29)					21 / 1245		1.86		(1.13,	3.06)			
	P-value (raw, adjusted) 0.46, 1.00										0.43, 1.00										0.02, 0.22						

^1^ Hazard Ratios (HR) and 95% Confidence Intervals (CI) among primary invasive cases; adjusted for age, study, BMI during referent year, parity, genetic ancestry, and SEER summary stage. Includes data from the 4-CBCS and SFBCS.

## Discussion

Based on ARTP results, *GPX3* was borderline statistically significantly associated with breast cancer risk for all women and for women with lower levels of NA ancestry specifically. *SEPP1* showed a statistically significant interaction by NA ancestry, with a strong association with breast cancer risk among women with higher NA ancestry. Some differences in association were observed by ER/PR tumor status. *GPX1, GPX3*, and *SELS* were significantly associated with ER-/PR+ tumors and *SELS* was significantly associated with ER+/PR- tumors. *GPX4* was significantly associated with survival among those with higher NA ancestry and *SEPW1* was marginally associated with survival among women in the low NA ancestry group. Although we hypothesized that DOBS would modify associations with selenoprotein genes, only one SNP in *GPX4*, *SELS*, and *TXNRD1* that interacted with DOBS remained statistically significantly associated with breast cancer after adjustment for multiple comparisons.

Among the genes evaluated in this study, only *GPX3* showed a borderline significant association overall as determined by the P_ARTP_, and *GPX3* rs8177447 was statistically significant after adjustment for multiple comparisons. This SNP is in high LD with rs3792797 and has been associated with Barrett’s Esophagus [29] (see table 5 for comparison of findings of this study to other information on SNPs). *GPX3* is one of multiple glutathione peroxidases, all of which are selenoproteins that play a role in catalyzing the reduction of hydrogen peroxides to minimize oxidative stress which can damage cells [10]. This enzyme acts as an efficient antioxidant in the plasma and has previously been linked to other diseases associated with oxidative stress [10]. Our study indicates that in addition to an important role in development of these cancers, *GPX3* may also play a role in the development and progression of breast cancer. 

**Table 5:** LD comparison of SNPs associated with breast cancer risk or with survival to other SNPs reported in literature as associated with cancer, serum markers, gene expression, or identified as being non-synonymous (NS) coding SNPs.

**Gene**	**SNP**	**Location/Coding type**	**Functional SNP**	**LD^1^ r^2^**
*GPX3*	rs8177447	intronic enhancer	rs4958872 [6,29]	0.67
			rs3792797 [6]	0.95
			rs3828599 [47]	NA
			rs3805435 [47]	NA
*GPX4*	rs2074451	3' downstream	rs713041 [3,40,42,48,49]	0.59
			rs2075710 [36]	0.27
			rs2074452 [36]	0.34
			rs757229 [42]	0.72
*SELS*	rs4965814	intronic	rs4965373 [40]	0.08
			rs34713741 [4]	0.001
*SEPP1*	rs230812	3' downstream	rs3877899[3,34,47,48] NS	0.31
			rs7579 [3,34,49]	0.34
			rs230813 [4,38]	1.00
			rs230819 [4,38]	0.70
			rs11959466 [35]	0.04
			rs13168440 [35]	0.19
			rs3797310 [36]	0.32
			rs12055266 [37,47]	0.32
	rs6865453	5' upstream	rs3877899 NS	0.14
		promoter	rs7579	0.96
		/regulatory region	rs230813	0.32
			rs230819	0.42
			rs11959466	0.12
			rs13168440	0.09
			rs3797310	1.00
			rs12055266	0.92
*SEPW1*	rs3786777	intronic enhancer		NA
*TXNRD1*	rs4964287	splicing regulation	rs1128446 [36]	0.71
		coding; synonymous	rs4964785 [36]	0.52
			rs7310505 [3]	0.08
*TXNRD2*	rs9606173	intronic enhancer	rs9605031 [40]	0.02
			rs1139793 [50] NS	0.07
			rs5992495 NS	0.006
			rs5748469 NS	0.003
	rs732262	intronic enhancer	rs9605031	0.11
			rs1139793 NS	0.13
			rs5992495 NS	0.12
			rs5748469 NS	0.18
	rs3788314	intronic	rs9605031	0.27
			rs1139793 NS	<0.001
			rs5992495 NS	0.28
			rs5748469 NS	0.18
	rs3788317	intronic	rs9605031	0.10
			rs1139793 NS	0.03
			rs5992495 NS	0.53
			rs5748469 NS	0.001
	rs5992493	intronic enhancer	rs9605031	0.05
			rs1139793 NS	0.12
			rs5992495 NS	1.00
			rs5748469 NS	0.12
	rs4333017	intronic	rs9605031	0.003
			rs1139793 NS	0.006
			rs5992495 NS	0.04
			rs5748469 NS	0.001

^1^ LD determined for SNPs using SNAP, from the Broad Institute, with the 1000 genome data option; in some instance LD results were not available (NA)

Other studies have indicated that glutathione peroxidases may be associated with breast cancer risk, specifically *GPX1* [14,30], a cytosolic antioxidant [31]. A meta-analysis of six case-control studies of the Pro198Leu polymorphism (rs1050450) in *GPX1*, did not see an association between breast cancer risk in Caucasians, although they did see a strong increased risk of breast cancer among African women [32]. Likewise, Cox and colleagues did not see an association between this SNP and breast cancer risk [33]. In a recent study by Meplan, *GPX1* rs1050450 was shown to interact with hormone therapy to alter risk of breast cancer [34]. Our study did not show an association with breast cancer risk and *GPX1*. We did however, detect an association between *GPX1* and ER-/PR+ breast tumors; however this represents a small group of women and could be a chance finding. 

*SEPP1* SNPs have been associated with a variety of cancers, including prostate [35,36], lung [2], and colorectal [3,37] cancer. Therefore, we evaluated three candidate *SEPP1* SNPs that have been associated with oxidative stress and cancer [6,38] and are in high LD with other functional SNPs (see Table 5). SEPP1 is the major selenoprotein in plasma, acting as a selenium transport protein [13]. SEPP1 has been shown to behave as an antioxidant [13] and estrogen has been shown to increase hepatic SEPP1 concentrations [39], providing biological support for the observed associations between *SEPP1* SNPs and cancer risk. Support for *SEPP1* as an antioxidant comes from earlier findings that in human plasma the SEPP1 protein is involved in the degradation of peroxynitrite, which plays a role in inflammatory toxicity [31]. Additionally, associations between serum selenium levels and thioredoxin reductase activity have been correlated with *SEPP1* rs3877899 [40], thereby establishing a further link between *SEPP1* and the antioxidant activities of selenoproteins. Our analysis failed to find an association between our candidate SNP, *SEPP1* rs3877899, that was previously linked to breast cancer [26] and is a non-synonymous coding SNP. In our analysis of two other *SEPP1* SNPs, rs230812 and rs6865453, we found an association with breast cancer risk among women with higher NA ancestry. *SEPP1* rs230812 is in high LD with rs230813 and rs230819 (see Table 5) which have been associated with oxidative stress [6,38]. *SEPP1* was the only significant gene associated with breast cancer risk among women with greater NA ancestry which was significantly different than the risk observed for women with low NA ancestry. In this study, a large percentage of women with greater NA ancestry were part of the Mexico City Breast Cancer Study and it is possible that differences in selenium levels in food could exist between those women and women in the United States. If women from Mexico had lower serum selenium it is possible that *SEPP1* could have a greater effect on risk.

We found that some selenoproteins were associated with tumor ER/PR status. Based on our analysis of gene P_ARTP_, *GPX1, GPX3*, and *SELS* were associated with ER-/PR+ tumor status, while *SELS* was also associated with ER+/PR- tumors. Additionally, several individual SNPs were associated with ER/PR status after adjusting for multiple comparisons. An earlier study found that glutathione peroxidase expression was associated with PR- status, as well as increased patient mortality [41]. The differences in results may be explained by the fact that the earlier study looked solely at expression levels of glutathione peroxidases, while our study looked at gene and SNP interactions without looking at expression of proteins. Other studies have shown links between selenoproteins and estrogen. GPX1 messenger RNA has been shown to be up-regulated in the presence of estrogen [42]. TXNRD1 has been shown to be an important modular of estrogen signaling through the estrogen receptor response elements [43]

We also observed associations between selenoprotein SNPs and survival. Notably, *GPX4* rs2074451 showed marginally significant interaction with NA ancestry and having a T allele was associated with decreased likelihood of dying from breast cancer among women with >28% NA ancestry. This is in agreement with study by Udler and colleagues [42], where they reported that *GPX4* rs757229 and rs713041 were associated with a greater risk of all-cause mortality after diagnosis with breast cancer. *GPX4* rs2074451 is highly correlated with these SNPs (see Table 5). Additionally, three SNPs in *TXNRD2* (rs3788314, rs3788317, and rs4333017) showed significant differences in survival by ancestry. TXNRD2 has been associated with oxidative stress and our previous analysis of dietary factors of oxidative stress found the strongest associations among women with higher NA ancestry [22]. Udler did not observe a significant association between any of the *TXNRD1* or *TXNRD2* SNPs and breast cancer survival [42].

Given their role as antioxidants and mediators of oxidative stress, we evaluated selenoprotein SNPs for interactions with DOBS and found that *GPX3* rs2070593, *GPX4* rs2074451, *SELS* rs9874, and *TXNRD1* rs17202060 showed significant interactions with DOBS after adjusting for multiple comparisons. Oxidative stress and high levels of reactive oxidative species have been suggested to play an important role in breast cancer development because free radicals damage DNA, thereby decreasing genomic integrity [44]. Our observed interactions with DOBS indicate that high DOBS may reduce breast cancer risk in individuals with high-risk genotypes. 

The selenoprotein genes analyzed in this study were selected due to previous studies reporting on their roles in regulating oxidative stress and/or carcinogenesis; however, the majority of the genes and SNPs had not been studied in relation to breast cancer. Table 5 compares SNPs associated with breast cancer risk and survival in our study, to those reported in the literature in alter cancer risk, influence oxidative stress, or influence gene expression. Selenium levels have been associated with breast cancer [45], along with SNPs in *GPX1* [14] and *GPX4* [46]. We did not find evidence for an association with breast cancer risk for these particular glutathione peroxidases, yet we found that *GPX3* was associated marginally with breast cancer risk and *SEPP1* was associated with risk among women with higher NA ancestry. *GPX4* was associated with breast cancer survival. Glutathione peroxidases carry out similar functions and have similar mechanisms; they contain a conserved catalytic triad of Sec, Gln, and Trp that acts by sequential oxidation and reduction of the Sec residue during catalysis [10]. The primary difference between the different glutathione peroxidases appears to be tissue distribution and cellular location; therefore, it is highly likely that multiple glutathione peroxidases participate in the antioxidant defense system against oxidative damage. 

Our study has two primary limitations. First, we only evaluated a subset of the known selenoproteins and did not evaluate any DIOs or SPS2s. Since these selenoproteins, along with others, play a role in limiting oxidative stress it is possible that they may be associated with breast cancer risk. Our second limitation was in our analysis of ER/PR status and survival where we were unable to include data from Mexico. While our study population was large, the sample sizes for the different ER/PR subtypes were small, thereby decreasing the statistical power of our analysis. Nevertheless, our study has multiple strengths: our analytic approach that evaluated the pathway as a whole, and our analysis of genes beyond individual SNPs via P_ARTP_. These strengths allowed us to show that both *GPX3* and *SEPP1* were associated with breast cancer; these associations warrant further study in other populations. An additional strength is our genetically admixed population that allowed us to evaluate associations across the spectrum of European to Native ancestry. 

While we observed few significant associations between selenoprotein genes and breast cancer risk, *GPX3* was marginally significant among women with lower NA ancestry and *SEPP1* was statistically significant among women with higher NA ancestry. Additionally, several genes were associated with ER/PR status. While we hypothesized that selenoprotein genes would interact with DOBS, only four SNPs significantly interacted with DOBS after adjustment for multiple comparisons. In conclusion, this study provides limited support for an association between selenoprotein genes and risk of breast cancer.

## Supporting Information

Table S1 **List of SNPs assessed.**
(DOCX)

Table S2 **MAF and HWE by level of Native American ancestry.**
(DOCX)
