# Supplementary material for: SEPP1 Influences Breast Cancer Risk among Women with Greater Native American Ancestry: The Breast Cancer Health Disparities Study
Source: PLoS One. 2013 Nov 20;8(11):e80554. doi: 10.1371/journal.pone.0080554 (PMC3835321; doi:10.1371/journal.pone.0080554)
Supplement: Table S1 — List of SNPs assessed. (DOCX) [file pone.0080554.s001.docx]

| Supplement Table S1. List of SNPs assessed | |  |  |  |  |  |  |  |  |
| --- | --- | --- | --- | --- | --- | --- | --- | --- | --- |
|  |  | Chromosome |  |  | Major/Minor | MAF | | FDR HWE *p* | |
| Gene | Alias | Location | Gene *P_ARTP_^1^* | dbSNP ID | Allele | NHW | HISP/NA | NHW | HISP/NA |
| *GPX1* | GSHPX1, MGC14399, MGC88245 | 3p21.3 | 0.80 | rs1800668 | C/T | 0.31 | 0.16 | 0.81 | 0.48 |
|  |  |  |  | rs3448 | C/T | 0.26 | 0.14 | 0.96 | 0.71 |
| *GPX2* | GI-GPx, GPRP, GSHPX-GI | 14q24.1 | 0.94 | rs10133290 | A/C | 0.21 | 0.21 | 1.00 | 0.72 |
|  | GSHPx-2 |  |  | rs11623705 | G/T | 0.12 | 0.09 | 0.96 | 0.92 |
|  |  |  |  | rs2737844 | C/T | 0.30 | 0.37 | 0.96 | 0.98 |
|  |  |  |  | rs2296327 | G/A | 0.21 | 0.24 | 0.78 | 0.59 |
| *GPX3* | GPx-P, GSHPx-3, GSHPx-P | 5q23 | 0.09 | rs8177447 | C/T | 0.17 | 0.12 | 0.86 | 0.43 |
|  |  |  |  | rs2070593 | G/A | 0.18 | 0.26 | 0.91 | 0.51 |
|  |  |  |  | rs3828599 | C/T | 0.25 | 0.34 | 0.93 | 0.51 |
| *GPX4* | MCSP, PHGPx, snGPx, snPHGPx | 19p13.3 | 0.74 | rs2074451 | G/T | 0.46 | 0.36 | 0.96 | 0.80 |
| *SEPP1* | SeP, SELP, Selenoprotein P |  | 0.16 | rs230812 | A/C | 0.48 | 0.34 | 0.03 | 0.94 |
|  |  | 5q31 |  | rs3877899 | C/T | 0.22 | 0.16 | 0.95 | 0.15 |
|  |  |  |  | rs6865453 | A/C | 0.28 | 0.47 | 0.95 | 0.25 |
| *SELS* | AD-015, ADO15, MGC104346 | 15q26.3 | 0.70 | rs9874 | A/G | 0.14 | 0.12 | 0.89 | 0.68 |
|  | MGC2553, SBBI8, SEPS1, VIMP |  |  | rs4965814 | T/C | 0.18 | 0.39 | 0.96 | 0.67 |
| *SEP15* |  | 1p31 | 0.37 | rs5859 | C/T | 0.21 | 0.16 | 0.96 | 0.69 |
|  |  |  |  | rs486133 | T/C | 0.20 | 0.14 | 1.00 | 0.47 |
|  |  |  |  | rs561104 | A/G | 0.42 | 0.44 | 0.96 | 0.71 |
|  |  |  |  | rs1407131 | T/C | 0.13 | 0.09 | 0.86 | 0.33 |
| *SEPN1* | FLJ24021, MDRS1, RSMD1 | 1p36.13 | 0.46 | rs718391 | C/G | 0.45 | 0.32 | 0.89 | 0.75 |
|  | RSS, SELN |  |  | rs2072749 | A/G | 0.26 | 0.22 | 0.97 | 0.38 |
|  |  |  |  | rs11247735 | A/G | 0.51 | 0.30 | 0.96 | 0.76 |
|  |  |  |  | rs4659382 | C/G | 0.26 | 0.21 | 0.96 | 0.50 |
|  |  |  |  | rs2294228 | T/G | 0.21 | 0.14 | 0.97 | 0.06 |
| *SEPW1* | selW | 19q13.3 | 0.97 | rs10412896 | T/C | 0.34 | 0.40 | 0.96 | 0.63 |
|  |  |  |  | rs3786777 | T/G | 0.51 | 0.45 | 0.62 | 0.84 |
|  |  |  |  | rs2042286 | C/T | 0.38 | 0.39 | 0.91 | 0.93 |
| *TXNRD1* | GRIM-12, MGC9145, TR, TR1 | 12q23-q24.1 | 0.29 | rs4964778 | C/G | 0.18 | 0.10 | 0.97 | 0.56 |
|  | TRXR1, TXNR |  |  | rs4964779 | T/C | 0.11 | 0.23 | 0.78 | 0.05 |
|  |  |  |  | rs4523760 | T/C | 0.23 | 0.14 | 0.96 | 0.88 |
|  |  |  |  | rs5018287 | G/A | 0.46 | 0.47 | 0.98 | 0.83 |
|  |  |  |  | rs4964287 | C/T | 0.31 | 0.21 | 0.86 | 0.43 |
|  |  |  |  | rs17202060 | C/T | 0.35 | 0.41 | 0.49 | 0.39 |
|  |  |  |  | rs7962759 | C/G | 0.20 | 0.10 | 0.91 | 0.43 |
| *TXNRD2* | SELZ, TR, TR-BETA, TR3, TRXR2 | 22q11.21 | 0.25 | rs5748469 | A/C | 0.65 | 0.39 | 0.96 | 0.38 |
|  |  |  |  | rs1044732 | A/G | 0.15 | 0.08 | 1.00 | 0.64 |
|  |  |  |  | rs3788305 | A/G | 0.47 | 0.40 | 1.00 | 0.30 |
|  |  |  |  | rs3788306 | T/C | 0.31 | 0.25 | 0.99 | 0.46 |
|  |  |  |  | rs2073750 | G/A | 0.23 | 0.31 | 0.96 | 0.74 |
|  |  |  |  | rs9606173 | A/T | 0.16 | 0.22 | 1.00 | 0.43 |
|  |  |  |  | rs5992493 | A/G | 0.17 | 0.14 | 0.96 | 0.86 |
|  |  |  |  | rs732262 | G/A | 0.09 | 0.18 | 0.86 | 0.86 |
|  |  |  |  | rs3788314 | G/A | 0.46 | 0.48 | 0.96 | 0.68 |
|  |  |  |  | rs3788317 | G/T | 0.23 | 0.25 | 0.96 | 0.68 |
|  |  |  |  | rs7410379 | G/A | 0.30 | 0.44 | 1.00 | 0.89 |
|  |  |  |  | rs756661 | T/C | 0.45 | 0.24 | 0.98 | 0.39 |
|  |  |  |  | rs17745445 | G/A | 0.14 | 0.08 | 0.96 | 0.70 |
|  |  |  |  | rs1978058 | C/T | 0.38 | 0.19 | 0.73 | 0.12 |
|  |  |  |  | rs8141691 | G/A | 0.37 | 0.46 | 0.96 | 0.75 |
|  |  |  |  | rs9306229 | C/T | 0.23 | 0.13 | 0.96 | 0.29 |
|  |  |  |  | rs4333017 | C/T | 0.13 | 0.08 | 0.96 | 0.70 |
|  |  |  |  | rs5746847 | C/T | 0.44 | 0.34 | 0.96 | 0.44 |
|  |  |  |  | rs9605030 | C/T | 0.14 | 0.09 | 0.99 | 0.51 |
|  |  |  |  | rs2020917 | C/T | 0.27 | 0.17 | 0.97 | 0.30 |
| ^1^Pathway P_ARTP_=0.69 | |  |  |  |  |  |  |  |  |
