# Supplementary material for: SEPP1 Influences Breast Cancer Risk among Women with Greater Native American Ancestry: The Breast Cancer Health Disparities Study
Source: PLoS One. 2013 Nov 20;8(11):e80554. doi: 10.1371/journal.pone.0080554 (PMC3835321; doi:10.1371/journal.pone.0080554)
Supplement: Table S2 — MAF and HWE by level of Native American ancestry. (DOCX) [file pone.0080554.s002.docx]

| Online Supplemental Table S2. MAF and HWE by level of Native American Ancestry | | | | | | | | | | |
| --- | --- | --- | --- | --- | --- | --- | --- | --- | --- | --- |
|  |  | 0 - 28% Native American Ancestry | | | 29 - 70% Native American Ancestry | | | 71 - 100% Native American Ancestry | | |
|  |  | Major/Minor Allele |  |  | Major/Minor Allele |  |  | Major/Minor Allele |  |  |
| Gene | SNP |  | MAF | FDR_HWE |  | MAF | FDR_HWE |  | MAF | FDR_HWE |
| *GPX1* | rs1800668 | C/T | 0.31 | 0.36 | C/T | 0.17 | 0.88 | C/T | 0.10 | 0.25 |
|  | rs3448 | C/T | 0.25 | 0.62 | C/T | 0.15 | 0.83 | C/T | 0.07 | 0.46 |
| *GPX2* | rs10133290 | A/C | 0.21 | 0.94 | A/C | 0.22 | 0.76 | A/C | 0.20 | 0.89 |
|  | rs11623705 | G/T | 0.12 | 0.89 | G/T | 0.10 | 0.89 | G/T | 0.08 | 0.75 |
|  | rs2737844 | C/T | 0.30 | 0.94 | C/T | 0.39 | 0.94 | C/T | 0.35 | 0.75 |
|  | rs2296327 | G/A | 0.21 | 0.52 | G/A | 0.24 | 0.52 | G/A | 0.24 | 0.75 |
| *GPX3* | rs8177447 | C/T | 0.17 | 0.29 | C/T | 0.13 | 0.77 | C/T | 0.05 | 0.06 |
|  | rs2070593 | G/A | 0.18 | 0.26 | G/A | 0.25 | 0.49 | G/A | 0.33 | 0.77 |
|  | rs3828599 | C/T | 0.26 | 0.77 | C/T | 0.34 | 0.49 | C/T | 0.36 | 0.77 |
| *GPX4* | rs2074451 | G/T | 0.46 | 0.87 | G/T | 0.37 | 0.87 | G/T | 0.33 | 0.87 |
| *SELS* | rs9874 | A/G | 0.14 | 0.45 | A/G | 0.13 | 0.78 | A/G | 0.06 | 0.78 |
|  | rs4965814 | T/C | 0.19 | 0.78 | T/C | 0.39 | 0.78 | T/C | 0.44 | 0.38 |
| *SEP15* | rs5859 | C/T | 0.21 | 0.88 | C/T | 0.17 | 0.88 | C/T | 0.11 | 0.88 |
|  | rs486133 | T/C | 0.20 | 0.88 | T/C | 0.15 | 0.88 | T/C | 0.09 | 0.88 |
|  | rs561104 | A/G | 0.42 | 0.88 | A/G | 0.43 | 0.88 | A/G | 0.48 | 0.88 |
|  | rs1407131 | T/C | 0.13 | 0.88 | T/C | 0.09 | 0.88 | T/C | 0.07 | 0.88 |
| *SEPN1* | rs718391 | C/G | 0.45 | 0.32 | C/G | 0.33 | 0.86 | C/G | 0.26 | 0.32 |
|  | rs2072749 | A/G | 0.26 | 0.86 | A/G | 0.22 | 0.17 | A/G | 0.20 | 0.51 |
|  | rs11247735 | A/G | 0.50 | 0.63 | A/G | 0.32 | 0.86 | A/G | 0.19 | 0.86 |
|  | rs4659382 | C/G | 0.26 | 0.86 | C/G | 0.21 | 0.17 | C/G | 0.19 | 0.28 |
|  | rs2294228 | T/G | 0.22 | 0.63 | T/G | 0.15 | 0.17 | T/G | 0.07 | 0.09 |
| *SEPP1* | rs230812 | A/C | 0.46 | 0.22 | A/C | 0.36 | 0.64 | A/C | 0.26 | 0.64 |
|  | rs3877899 | C/T | 0.23 | 0.69 | C/T | 0.17 | 0.64 | C/T | 0.07 | 0.05 |
|  | rs6865453 | A/C | 0.28 | 0.84 | A/C | 0.44 | 0.64 | C/A | 0.38 | 0.82 |
| *SEPW1* | rs10412896 | T/C | 0.34 | 0.55 | T/C | 0.40 | 0.33 | T/C | 0.40 | 0.50 |
|  | rs3786777 | G/T | 0.49 | 0.12 | T/G | 0.44 | 0.50 | T/G | 0.47 | 0.50 |
|  | rs2042286 | C/T | 0.39 | 0.50 | C/T | 0.40 | 0.50 | C/T | 0.35 | 0.12 |
| *TXNRD1* | rs4964778 | C/G | 0.18 | 0.86 | C/G | 0.11 | 0.86 | C/G | 0.05 | 0.93 |
|  | rs4964779 | T/C | 0.11 | 0.86 | T/C | 0.21 | 0.59 | T/C | 0.34 | 0.97 |
|  | rs4523760 | T/C | 0.23 | 0.86 | T/C | 0.15 | 0.92 | T/C | 0.07 | 0.92 |
|  | rs5018287 | G/A | 0.46 | 0.98 | G/A | 0.47 | 0.86 | G/A | 0.49 | 0.86 |
|  | rs4964287 | C/T | 0.31 | 0.92 | C/T | 0.22 | 0.97 | C/T | 0.14 | 0.93 |
|  | rs17202060 | C/T | 0.35 | 0.10 | C/T | 0.41 | 0.59 | C/T | 0.45 | 0.93 |
|  | rs7962759 | C/G | 0.20 | 0.86 | C/G | 0.11 | 0.92 | C/G | 0.05 | 0.92 |
| *TXNRD2* | rs5748469 | C/A | 0.36 | 0.99 | A/C | 0.41 | 0.92 | A/C | 0.24 | 0.99 |
|  | rs1044732 | A/G | 0.15 | 0.99 | A/G | 0.09 | 0.99 | A/G | 0.04 | 0.99 |
|  | rs3788305 | A/G | 0.48 | 0.99 | A/G | 0.42 | 0.99 | A/G | 0.30 | 0.99 |
|  | rs3788306 | T/C | 0.31 | 0.99 | T/C | 0.26 | 0.99 | T/C | 0.19 | 0.99 |
|  | rs2073750 | G/A | 0.23 | 0.99 | G/A | 0.30 | 0.88 | G/A | 0.34 | 0.99 |
|  | rs9606173 | A/T | 0.16 | 0.99 | A/T | 0.22 | 0.88 | A/T | 0.24 | 0.92 |
|  | rs5992493 | A/G | 0.17 | 0.99 | A/G | 0.15 | 0.99 | A/G | 0.10 | 0.99 |
|  | rs732262 | G/A | 0.09 | 0.88 | G/A | 0.17 | 0.92 | G/A | 0.23 | 0.99 |
|  | rs3788314 | G/A | 0.46 | 0.99 | G/A | 0.49 | 0.99 | G/A | 0.45 | 0.88 |
|  | rs3788317 | G/T | 0.23 | 0.99 | G/T | 0.25 | 0.92 | G/T | 0.27 | 0.99 |
|  | rs7410379 | G/A | 0.30 | 0.99 | G/A | 0.42 | 0.88 | A/G | 0.49 | 0.99 |
|  | rs756661 | T/C | 0.44 | 0.99 | T/C | 0.25 | 0.99 | T/C | 0.14 | 0.92 |
|  | rs17745445 | G/A | 0.15 | 0.99 | G/A | 0.08 | 0.99 | G/A | 0.03 | 0.99 |
|  | rs1978058 | C/T | 0.37 | 0.92 | C/T | 0.21 | 0.99 | C/T | 0.09 | 0.99 |
|  | rs8141691 | G/A | 0.37 | 0.99 | G/A | 0.45 | 0.88 | A/G | 0.46 | 0.99 |
|  | rs9306229 | C/T | 0.23 | 0.99 | C/T | 0.14 | 0.99 | C/T | 0.07 | 0.99 |
|  | rs4333017 | C/T | 0.13 | 0.99 | C/T | 0.09 | 0.88 | C/T | 0.03 | 0.03 |
|  | rs5746847 | C/T | 0.43 | 0.99 | C/T | 0.33 | 0.88 | C/T | 0.37 | 0.99 |
|  | rs9605030 | C/T | 0.14 | 0.99 | C/T | 0.10 | 0.99 | C/T | 0.06 | 0.99 |
|  | rs2020917 | C/T | 0.27 | 0.99 | C/T | 0.19 | 0.99 | C/T | 0.09 | 0.93 |
